# The Fear of COVID-19 Scale: Psychometric Properties of the Ethiopian Amharic Version

**DOI:** 10.1007/s11469-020-00448-0

**Published:** 2020-12-03

**Authors:** Aman Sado Elemo, Seydi Ahmet Satici, Mark D. Griffiths

**Affiliations:** 1 Psychology Department, İstanbul Gelisim University, İstanbul, Turkey; 2 Department of Psychological Counselling, Artvin Coruh University, Artvin, Turkey; 3 International Gaming Research Unit, Psychology Department, Nottingham Trent University, Burton Street, Nottingham, NG1 4FQ UK

**Keywords:** COVID-19, Coronavirus-2019, Fear of COVID-19, Loneliness, Ethiopia, Amharic

## Abstract

Fear is an adaptive response that alerts individuals to the presence of a danger or threat. However, in the context of the current novel coronavirus disease 2019 (COVID-19) pandemic, the fear experienced could be intense because the number of victims of the virus is continuously increasing globally and is inducing severe mental health concerns. The seven-item Fear of COVID-19 Scale (FCV-19S) assesses the severity of fear related to COVID-19 and has already been validated in many languages. The present study evaluated the psychometric properties of the Amharic (Ethiopian) version of the FCV-19S. An online survey including the Amharic versions of the FCV-19S and the six-item UCLA Loneliness Scale (ULS-6) was administered to 307 Amharic-speaking participants using convenience sampling. The participants’ age ranged between 18 and 70 years. In the evaluation process, confirmatory factor analysis, Item Response Theory, concurrent validity, and reliabilities (Cronbach’s alpha, McDonald’s omega, Guttman’s lambda, and composite reliability) of the Amharic version of the FCV-19S were performed. The uni-dimensional structure of the FCV-19S was confirmed and the Amharic version of the FCV-19S had strong psychometric properties. All reliability coefficients of the Amharic FCV-19S were satisfactory, with sound concurrent validity shown by significant and positive correlations with loneliness. The results indicate that the FCV-19S can be used in research to assess the fear of COVID-19 among Amharic-speaking populations.

The novel coronavirus desease-19 (COVID-19) outbreak has been deemed a global health emergency by the World Health Organization (WHO 2020a). As it has spreads across the world, the pandemic has induced worries and fears increasing the risks of mental health problems. Internationally, the number of confirmed coronavirus cases has continued to increase and has claimed hundreds of thousands of lives. At the time of writing (November 2020), the number of internationally confirmed COVID-19 cases was over 55 million and over 1.32 million deaths (WHO 2020a).

In the case of Africa, although the continent accounts for a small fraction of the total global COVID-19 cases, the increase in the number of confirmed cases is still alarming since the first case was recorded in mid-February 2020. By mid-November 2020, the number of confirmed COVID-19 cases had exceeded 1.9 million resulting in more than 46,800 deaths in the continent (Africa Center for Disease Control 2020). South Africa, Morocco, Egypt, Ethiopia, and Nigeria were the top five countries with high COVID-19 cases by the end of October 2020. Unless early detection and a robust system of preventing the spread of the virus are implemented, the COVID-19 outbreak may claim more lives in populous countries like Ethiopia. By mid-November 2020, Ethiopia had reported over 101,700 COVID-19 cases and over 1550 deaths (Information Network Security Agency 2020).

As part of its response to minimize the spread of COVID-19, Ethiopia has introduced various measures. These include delaying of national elections, declaration of national state of emergency, closing schools, enforcing physical distancing and facemasks in public, and a mandatory quarantine (Ethiopia UNHCR Global Focus 2020). These measures were introduced in early 2020 and scaled up a coordinated national response following the first case of coronavirus. However, because there was no national lockdown (Oqubay 2020), and some of the measures were relaxed after June 19, 2020 (Anadolu Agency 2020), there is a fear of uncontrolled large-scale community transmission of the virus. Despite the strict measures implemented, the number of new daily COVID-19 cases continued to increase and peaked at 1829 cases on August 21, 2020 (Daily New COVID-19 Cases in Ethiopia, Worldometers 2020). Furthermore, emerging study results suggest that stringent public health measures to inhibit the spread of the virus can result in isolation, financial insecurities, and disrupt daily life activities, which may also have negative consequences to psychological wellbeing (Galea et al. 2020; Satici et al. 2020). Being away from school and workplaces, possibilities of losing jobs, increased family responsibilities, and adjusting to these changes in life also have the potential to exacerbate pre-existing mental health problems (Fegert et al. 2020).

Public health measures introduced to curb the spread of virus may also result in less comfortable everyday living and result in negative psychosocial consequences (e.g., stigma). These situations demand individuals to make changes in their habits and behavioral patterns. Such forced changes and prohibitions of going to school, workplaces, or other already established patterns of living due to the COVID-19 may increase frustrations and anxieties. Mamun and Griffiths (2020) noted that social distancing, isolation, and quarantine can trigger a wide range of psychological responses including sadness, worry, fear, anger, annoyance, frustration, guilt, helplessness, loneliness, and nervousness.

As the number of COVID-19 cases increases, the possibilities of being exposed to this life-threatening virus also increase. This would likely increase uncertainties and thought ruminations about contracting the virus. Individuals experience fear of being infected by the virus and worry about the health of their loved ones (Satici et al. 2020). Therefore, regardless of exposure, individuals may experience fear of falling sick or dying. Studies have reported a high prevalence of psychological distress during the COVID-19 pandemic (e.g., Ahorsu et al. 2020; Satici et al. 2020). There could also be distrust of others as the virus spreads. Individuals may end up losing employment and experiencing financial losses, which would in turn heighten fear, frustrations, and uncertainty among individuals. These in turn may intensify negative emotional experiences and increase irrational reactions to the situations that demand conscious reactions that could minimize chances of being infected by the virus.

During the COVID-19 pandemic, there appears to have been a substantial increase in domestic violence, child abuse, and sexual assault. For instance, the WHO has also highlighted an increase in levels of violence occurring in the home, including violence against children, intimate partner violence, and violence against older individuals in several countries affected by the COVID-19 pandemic (WHO 2020b). During a 2-month period during the pandemic in Addis Ababa (Ethiopia), a police investigation reported 101 child sexual assault cases, with some of the attacks coming from family members (BBC News Afaan Oromoo 2020b). Emerging evidence also indicates that during the COVID-19 pandemic, “stay home” regulations have resulted in heightened rates of physical, sexual, and emotional intimate partner violence (Thakur and Jain 2020; Closson et al. 2020).

Additionally, in extreme cases, COVID-19-related suicides have been reported (e.g., Bhuiyan et al. 2020; Griffiths and Mamun 2020; Mamun and Griffiths 2020). Since the start of the COVID-19 pandemic, the WHO (2020b) has also given warning about the potential increase of self-harm or suicidal behaviors, loneliness, depression, and drug use. Arguably, the first COVID-19-related suicide case in Ethiopia was a 25-year-old woman who believed she was infected by the virus and was kept isolated from 36 passengers on a bus, who committed suicide by hanging herself using her scarf on June 2 (2020) in a room where she was quarantined alone (BBC News Afaan Oromoo 2020a). There are many COVID-19-related reasons that may contribute to suicidal behavior including (i) fear about one’s own health and the health of loved ones; (ii) fear of being threatened, ostracized, discriminated against, and stigmatized by others in the local community due to having (or thinking the individual has) the virus; (iii) pre-existing psychological issues; (iv) financial problems as a result of the COVID-19 measures (e.g., being unable to earn money during lockdowns, losing a job); or (v) lack of psychological readiness to embrace the crisis and move forward (Bhuiyan et al. 2020; Dsouza et al. 2020; Griffiths and Mamun 2020; Mamun and Griffiths 2020).

To reduce COVID-19 transmission and to manage its adverse psychological consequences, it is necessary to explore associated psychological consequences such as fear, anxiety, and stress. Attempts to introduce and implement psychological interventions as early as possible could lead to favorable changes in the psychological wellbeing of individuals. In recognition of the potential psychological impact, a number of new psychometric instruments have been developed to explore on psychological consequences of the virus including the COVID Stress Scale (Taylor et al. 2020), Coronavirus Anxiety Scale (Lee 2020), and the Fear of COVID-19 Scale (Ahorsu et al. 2020). The Fear of COVID-19 Scale developed by Ahorsu et al. (2020) assesses individual’s fear of COVID-19 and has been validated in different languages including English (Winter et al. 2020), Persian (Ahorsu et al. 2020), Turkish (Satici et al. 2020), Italian (Soraci et al. 2020), Arabic (Alyami et al. 2020), Bangla (Sakib et al. 2020), Russian (Reznik et al. 2020), Spanish (Broche-Pérez et al. 2020), Hebrew (Bitan et al. 2020), Japanese (Masuyama et al. 2020), Vietnamese (Nguyen et al. 2020), Greek (Tsipropoulou et al. 2020), Chinese (Chi et al. 2020), Malay (Pang et al. 2020), and French (Mailliez et al. 2020).

Translating, adapting, and validating the Fear of COVID-19 Scale (FCV-19S) may help to understand the level of fear of COVID-19 among the general population, test the efficiency of psychological interventions that may facilitate managing the psychological impacts of the pandemic, and help stimulate cross-cultural research. However, there is lack of psychometrically valid measures of fear of the COVID-19 in Ethiopia. Therefore, adapting and examining the psychometric characteristics of the Amharic version of the FCV-19S could be timely and valuable. Therefore, the present study adapted and validated the scale among an Amharic-speaking population and evaluated the scale’s psychometric properties. In relation to concurrent validity, it was hypothesized that fear of COVID-19 would be positively associated with loneliness.

## Method

### Adaptation of the FCV-19S to Amharic

The English version of the fear of COVID-19 Scale was translated to Amharic through backward and forward translations by two independent bilingual doctoral candidates (who were not authors of the research) but volunteers by using the parallel blind technique (Behling and Law 2000). The translators were knowledgeable about the content areas of the construct. The translated items were back translated, and necessary revisions were made in terms of their semantic, idiomatic, and conceptual equivalence. Items were reviewed to resolve ambiguities and discrepancies between the translations. Clarity and understandability of the items were checked following the procedures of cross-cultural psychological instruments’ adaptations suggested by Borsa et al. (2012). Consequently, the final Amharic version of the FCV-19S was piloted to check the understandability of the items and clarity of the instructions. The Amharic version of the FCV-19S was then finalized for the evaluation of the psychometric properties of the scale.

### Participants and Procedure

A cross-sectional study was conducted to examine the psychometric properties of the Amharic version of the Fear of COVID-19 Scale using online survey during May 21–May 25, 2020. The target population was the Amharic-speaking population which was completed via an online survey link prepared on *Google Forms.* The questions included items related to demographic information, fear of COVID-19, and loneliness. The survey was distributed on social media through the support of public figures followed by more than 5000 people, via e-mail to e-mails of academicians’ associations, and *WhatsApp* through *WhatsApp* groups of Ethiopians in the country and abroad.

Participants were asked about their willingness to take part in the study, their age, their gender, their country of residence, their knowledge of someone infected by the virus, and what preventive COVID-19 behaviors they personally engaged in. A total of 307 participants anonymously and voluntarily participated in an online survey using convenience sampling. These participants have provided their consent to participate in the study (see the “Ethics” section) and their age ranged between 18 and 70 years (*M* = 30.9, SD = 7.96) and 18.9% were female (*n* = 58). Most of the participants were young (*n* = 232, 75.6%) and 24% of the participants were middle-aged. Just under two-thirds of the sample were living in Ethiopia (62.87%) while 37.13% participants lived in other countries including Turkey, the USA, Saudi Arabia, Canada, Belgium, Kenya, Australia, Egypt, Germany, Hungary,the Netherlands, Norway, Qatar, Switzerland, and the United Arab Emirates. Detailed information about the participants is presented in Table 1.

**Table 1:** Participant demographics (*N* = 307)

Demographics		*n*	%
Gender	Male	249	81.1
	Female	58	18.9
Country of residence	Ethiopia	193	62.87
	Abroad	114	37.13
Region in Ethiopia	Addis Ababa	64	33.16
	Oromia	90	46.63
	Amhara	13	6.74
	SNNPR	12	6.22
	Tigray	4	2.07
	Harari	4	2.07
	Dire Dawa	3	1.55
	Somali	3	1.55
Relationship status	Single	141	45.9
	Married	166	54.1
Who they live with	Family	183	59.61
	Alone	70	22.80
	Friend(s)	54	17.59
Knowledge of someone infected with COVID-19	Yes	36	11.73
	No	271	88.27
Preventive measures taken against COVID-19	Wearing face masks	230	86.2
	Handwashing and sanitization	305	89.7
	Physical distancing	243	71.5
	Staying at home	158	46.5

### Measures

#### Fear of COVID-19 Scale (FCV-19S)

The FCV-19S is a uni-dimensional scale that assesses the fear of COVID-19 and was developed by Ahorsu et al. (2020). The instrument comprises seven items (e.g., “My heart races or palpitates when I think about getting coronavirus-19”) which are responded to on a 5-point Likert scale from 1 (*strongly disagree*) to 5 (*strongly agree*). The scores that can be obtained from the FCVS-19 vary between 7 and 35, and higher scores indicate greater fear of COVID-19. The original version of the Fear of COVID-19 Scale was adapted to Amharic through backward and forward translations by two independent bilingual doctoral candidates who were not authors of the research but volunteers by using the parallel blind technique (Behling and Law 2000). Clarity and understandability of the items were checked before they were made ready to be administered for the validation study by following the procedures of cross-cultural psychological instruments’ adaptations by Borsa et al. (2012).

#### The UCLA Loneliness Scale (ULS-6)

The ULS is a short form of the 20-item Revised UCLA Loneliness Scale (Russell et al. 1980) that assesses subjective feelings of loneliness (Neto 2014). The ULS-6 comprises six items that individuals are asked to rate their agreement with, of which five items (e.g., “How often do you feel that people are around you but not with you?”) were worded negatively and one item worded positively (Neto 2014). All items were scored on a 4-point scale ranging from 1 (*never*) to 4 (*often*). The internal consistency of the Amharic version of the ULS-6 in this study was .77. Its brevity, relevance in assessing loneliness and social isolation, and popularity were among the reasons in using the scale in the study. Loneliness has also been associated with various aspects of fear in previous research (e.g., Geukens et al. 2020; Jakobsson and Hallberg 2005; Rossi et al. 2020; Whitehead and Whitehead 2010).

### Ethics

The study was approved by the first author’s university ethics committee. Informed consent was obtained from participants through which they could make a knowledgeable decision on their willingness to participate in the study and complete the online survey. They were informed that they had the right to withdraw from the study at any time. Overall, the study procedures followed in the study were in accordance with the Helsinki Declaration of 1975, as revised in 2000.

### Data Analysis

First, descriptive statistics (means, standard deviations, variance, skewness, and kurtosis) of the FCV-19S were examined. The construct validity of the scale was then tested with confirmatory factor analysis (CFA) using maximum likelihood estimation. The following indexes and criteria are used in CFA: standardized root mean square residual (SRMR) was below .08 and comparative fit index (CFI), goodness of fit (GFI), and incremental fit index (IFI) were above .90 (Hu and Bentler 1999; Kline 2015). Item Response Theory (IRT Chalmers 2012) was utilized to evaluate the discrimination, difficulty, and informativeness of the FCV-19S. Consequently, the item characteristic curve (ICC) and Graded Response Model (GRM) were examined. Relationships between FCV-19S and loneliness were examined for concurrent validity. Inter-item correlations were also checked. Finally, Cronbach’s alpha, McDonald’s omega, Guttman’s lambda, and composite reliability were examined to evaluate the reliability of the FCV-19S. All analysis was carried out using IBM SPSS Statistics 22, AMOS 24, JASP 0.11.1, and Stata 14.2.

## Results

Analysis of the demographic data indicated that over one-tenth of the participants reported that they knew someone who had been infected with COVID-19 (11.7%). To minimize the spread of the virus, participants most frequently engaged in behaviors included handwashing and use of sanitizers (89.7%), wearing facemasks (86.2%), physical distancing (71.5%), and staying at home (46.5%). During the online survey, the participants reported that they were living with family (59.61%), living alone (22.80%), or living with one or more friends (17.59%).

Descriptive statistics (mean, SD, variance, skewness, and kurtosis) of the FCV-19S are presented in Table 2. All items had skewness and kurtosis values within the ± 1.5 range, indicating that they were normally distributed based on guidelines by Byrne and Campbell (1999). Fear of COVID-19 of those living in the country (*M* = 21.65, SD = 5.58) and not living in the country (*M* = 20.79, SD = 5.78) was compared and no significant difference was found (*t*[305] = 1.28, *p* = .19). After descriptive statistics, the construct validity of the FCV-19S was tested using CFA. The uni-dimensional structure of the FCV-19S fitted well with the data: CMID = 82.91, df = 14, CFI = .93, GFI = .92, NFI = .92, IFI = .93, SRMR = .059. Factor loadings ranged from .447 to .890 and were statistically significant (see Table 2). Results of the IRT are presented in Table 3. As shown in Table 2, all *α* values were higher than 1.0. Therefore, IRT results indicated that the FCV-19S has the appropriate item difficulty and the ability to discriminate between those who perform and those who do not.

**Table 2:** Descriptive statistics for the Fear of COVID-19 Scale

Item	Factor loading	Mean (SD)	CI	Variance	Skewness	Kurtosis
Item 1	.447**	4.23 (.87)	4.12–4.32	.76	− 1.32	1.68
Item 2	.465**	4.02 (1.02)	3.89–4.13	1.04	− 1.15	.86
Item 3	.811**	2.27 (1.04)	2.15–2.38	1.09	.43	− .56
Item 4	.678**	2.89 (1.15)	2.75–3.02	1.33	.06	− .93
Item 5	.721**	3.20 (1.19)	3.06–3.32	1.44	− .22	− 1.01
Item 6	.890**	2.21 (1.05)	2.09–2.32	1.11	.47	− .57
Item 7	.835**	2.53 (1.14)	2.41–2.66	1.32	.23	− .91

**p < .001. *SD*, standard deviation; *CI*, confidence interval

**Table 3:** Item Response Theory estimates for the Amharic FCV-19S

Item	Item parameter estimates				
	*α*	*b*_1_	*b*_2_	*b*_3_	*b*_4_
1	1.06	− 5.21	− 2.73	− 2.19	.36
2	1.09	− 3.77	− 2.16	− 1.54	.67
3	2.98	− .69	.30	1.36	2.39
4	1.92	− 1.57	− .20	.64	1.93
5	2.08	− 1.73	− .52	.10	1.45
6	5.11	− .52	.29	1.28	2.14
7	3.21	− .84	.07	.89	2.01

After IRT, the concurrent validity of the FCV-19S was tested by examining its correlation with loneliness. In conditions of limited contact, lack of desirable social interaction, and absence of someone to turn to during the COVID-19 pandemic, loneliness becomes a risk factor. Therefore, it was hypothesized that loneliness would correlate positively with fear of COVID-19. Results of the analysis showed that the FCV-19S score was significantly and positively associated with loneliness (*r* = .50, *p* < .001). Concurrent validity was supported by inter-item correlations (Table 4). Therefore, a positive and significant relationship between all items was observed (ranging between .30 and .76).

**Table 4:** Inter-item Pearson’s correlation matrix and corrected item-total correlations

Item	Item 1	Item 2	Item 3	Item 4	Item 5	Item 6	Item 7	Corrected item-total correlations
Item 1	–							.47
Item 2	.41**	–						.49
Item 3	.30**	.36**	–					.71
Item 4	.46**	.40**	.56**	–				.67
Item 5	.31**	.43**	.56**	.50**	–			.68
Item 6	.35**	.36**	.76**	.56**	.63**	–		.77
Item 7	.39**	.36**	.64**	.58**	.62**	.75**	–	.76

**p < .001

Different types of reliability of the Amharic FCV-19S were then assessed (i.e., Cronbach’s alpha, McDonald’s omega, Guttman’s lambda, and composite reliability were investigated). The reliability results indicated that the Cronbach’s alpha (*α* = .873), McDonald’s omega (*ω* = .876), Guttman’s lambda (*λ*6 = .873), and composite reliability (CR = .872) were all very good. Finally, the average variance extracted (AVE) was found to be .51, indicating acceptable convergent reliability.

## Discussion

The present study evaluated the psychometric properties of the Amharic version of the FCV-19S (see Appendix) among a general Amharic-speaking population both in and outside Ethiopia. This study is important not only for the assessment of COVID-19-related fear but also for the better understanding of vulnerability to psychological and physical illness due to the virus. During the pandemic, the increased tendency to be affected as much by the mental health than the infection itself (Reardon 2015) could be reversed if validated measures are employed as part of psychosocial intervention to minimize the spread of virus.

Overall, the results of the present study provide further support for the validity and reliability of the Fear of COVID-19 Scale, demonstrating good reliabilities, good concurrent validity, and acceptable construct validity for the Amharic version among Ethiopian populations. The results of the confirmatory factor analysis fitted the data very well and confirmed the scale’s uni-dimensional factor structure. This is consistent with the past analyses of this scale (e.g., Ahorsu et al. 2020; Sakib et al. 2020; Satici et al. 2020; Soraci et al. 2020; Winter et al. 2020). In addition, the IRT results presented similar findings to the Turkish FCV-19S (Satici et al. 2020) indicating that item difficulties and features of the Amharic FCV-19S as being appropriate.

Cronbach’s alpha for this uni-dimensional model was .87. Consequently, the internal consistency in the Amharic version of the FCV-19S is the same or very similar to other studies such as the English version (*α* = .84 to .86; Winter et al. 2020), the Italian version (*α* = .87; Soraci et al. 2020), the Hebrew version (*α* = .86; Bitan et al. 2020), the Russian version (*α* = .80; Reznik et al. 2020), the Bangla version (*α* = .87; Sakib et al. 2020), the Persian version (*α* = .82; Ahorsu et al. 2020), the Turkish version (*α* = .85, Satici et al. 2020), the Greek version (*α* = .87; Tsipropoulou et al. 2020), the Arabic version (*α* = .88; Alyami et al. 2020), the Malay version (*α* = .89; Pang et al. 2020), the Spanish version (*α* = .86; Broche-Pérez et al. 2020), and the French version (*α* = .87; Mailliez et al. 2020). In addition to the Cronbach’s alpha, other reliability coefficients of the FCV-19S (McDonald’s omega, Guttman’s lambda, and composite reliability) were found to be very good. In addition, having an AVE above .50 and a corrected item-total correlation of above .40 provided additional evidence for the reliability.

In this study, and as expected, the fear of COVID-19 pandemic was found to correlate positively with loneliness. One possible explanation could be related to the measures in place at the time of the study (e.g., stay home orders, physical distancing) that may have increased loneliness, potential stigma, and social disconnection among many people. This result is consistent with the research findings of Killgore et al. (2020) who reported loneliness was a critical mental health concern during the social isolation efforts to minimize the spread of the virus during the COVID-19 pandemic. Therefore, during the pandemic, spending more time alone, quarantine, and other measures that have been implemented to minimize the spread of the virus may bring about social isolation. Such measures may have elevated the level of loneliness among the study participants. Increased levels of loneliness, harmful alcohol and drug use, and self-harm or suicidal behavior have also been noted as being among the consequences due to the psychological impacts of the COVID-19 (Mamun and Griffiths 2020; WHO 2020a).

Although there are important findings related to the psychometric properties of the Fear of COVID-19 Scale, the present study has several limitations that need to be taken into consideration when interpreting the findings. First, conducting a survey with self-report measures entails potential bias due to socially desirable responses. Future studies should aim to utilize other methodologies that would enable a more in-depth analysis. Second, although the online survey used to collect data in the present study was helpful in reaching Amharic-speaking participants in both Ethiopia and other countries, this convenience sampling approach is unlikely to be representative of Amharic-speaking populations, especially those who are not able to access the internet. With convenience sampling, differences in the percentages of male and female participants are to be expected, but a large percentage of the study sample comprised male participants. Moreover, gender gap in the workforce due to limited opportunities for women’s education (World Bank 2019) together with high costs of internet might have contributed to gender differences in participating in the present study. This is likely to affect the generalizability of the findings. Future studies are encouraged to include more equally balanced percentages of male and female participants. Notwithstanding these limitations, the results of the present study provided support for the utility of the Amharic version of the FCV-19S and highlighted its potential to be used in clinical and research settings in the context of Amharic-speaking populations.

## Figures and Tables

**Figure:**
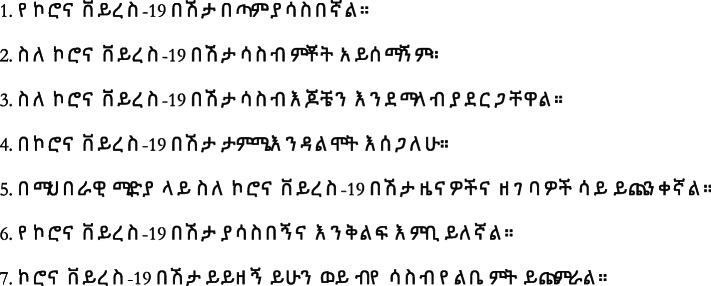
Amharic version of the Fear of the COVID-19 Scale

## References

[CR1] Africa Center for Disease Control (2020). Coronavirus Disease 2019. Retrieved November 28, 2020, from: https://africacdc.org/covid-19/

[CR2] Ahorsu, D. K., Lin, C. Y., Imani, V., Saffari, M., Griffiths, M. D., & Pakpour, A. H. (2020). The Fear of COVID-19 Scale: Development and initial validation. *International Journal of Mental Health and Addiction*. 10.1007/s11469-020-00270-8. 10.1007/s11469-020-00270-8 PMC7100496 32226353

[CR3] Alyami, M., Henning, M., Krägeloh, C. U., & Alyami, H. (2020). Psychometric evaluation of the Arabic version of the Fear of COVID-19 Scale. *International Journal of Mental Health and Addiction*. Advance online publication. 10.1007/s11469-020-00316-x. 10.1007/s11469-020-00316-x PMC7229877 32427217

[CR4] Anadolu Agency News (2020). Ethiopia confirms first coronavirus case. March 13. Retrieved November 28, 2020, from: https://www.aa.com.tr/en/africa/ethiopia-confirms-first-coronavirus-case/1765130

[CR5] BBC News Afaan Oromoo. (2020a). Arbaa Mincitti dubartiin shakkii COVID-19’n adda baafamte of ajjeefte. June 2. Retrieved November 28, 2020, from: https://bbc.in/37zNGPj.

[CR6] BBC News Afaan Ormoo. (2020b). Miidhaa daa’immanii: Dhimmi daa’imman 101 magaala Finfinnee keessatti gudeedaman rifannaa uume. June 4. Retrieved November 28, 2020, from: https://www.bbc.com/afaanoromoo/oduu-52924184.

[CR7] Behling, O., & Law, K. S. (2000). *Translating questionnaires and other research instruments: problems and solutions*. Thousand Oaks: SAGE. 10.4135/9781412986373.

[CR8] Bhuiyan, A. I., Sakib, N., Pakpour, A., Griffiths, M. D., & Mamun, M. A. (2020). COVID-19-related suicides in Bangladesh due to lockdown and economic factors: Case study evidence from media reports. *International Journal of Mental Health and Addiction*. 10.1007/s11469-020-00307-y. 10.1007/s11469-020-00307-y PMC7228428 32427168

[CR9] Bitan, D. T., Grossman-Giron, A., Bloch, Y., Mayer, Y., Shiffman, N., & Mendlovic, S. (2020). Fear of COVID19 scale: psychometric characteristics, reliability and validity in the Israeli population. *Psychiatry Research, 289*, 113100. 10.1016/j.psychres.2020.113100. 10.1016/j.psychres.2020.113100 PMC7227556 32425276

[CR10] Borsa, J. C., Damásio, B. F., & Bandeira, D. R. (2012). Adaptação e validação de instrumentos psicológicos entre culturas: Algumas considerações. *Paidéia (Ribeirão Preto), 22*(53), 423–432. 10.1590/s0103-863x2012000300014.

[CR11] Broche-Pérez, Y., Fernández-Fleites, Z., Jiménez-Puig, E., Fernández-Castillo, E., & Rodríguez-Martin, B. C. (2020). Gender and fear of COVID-19 in a Cuban population sample. *International Journal of Mental Health and Addiction*, Advance online publication. 10.1007/s11469-020-00343-8. 10.1007/s11469-020-00343-8 PMC7292241 32837428

[CR12] Byrne, B. M., & Campbell, T. L. (1999). Cross-cultural comparisons and the presumption of equivalent measurement and theoretical structure: A look beneath the surface. *Journal of Cross-Cultural Psychology, 30*(5), 555–574. 10.1177/0022022199030005001.

[CR13] Chalmers, R. P. (2012). mirt: A multidimensional item response theory package for the R environment. *Journal of Statistical Software, 48*(6), 1–29. 10.18637/jss.v048.i06.

[CR14] Chi, X., Chen, S., Chen, Y., Chen, D., Yu, Q., Guo, T., et al. (2020). *Psychometric evaluation of the Fear of COVID-19 Scale among Chinese population*. 10.31234/osf.io/t5jne. 10.1007/s11469-020-00441-7 PMC7799163 33456407

[CR15] Closson, K., Lee, M., Gibbs, A., & Kaida, A. (2020). When home is not a safe place: Impacts of social distancing directives on women living with HIV. *AIDS and Behavior*. Advance online publication*, 24*, 3017–3019. 10.1007/s10461-020-02941-y. 10.1007/s10461-020-02941-y PMC7266420 32488553

[CR16] Dsouza, D. D., Quadros, S., Hyderabadwala, Z. J., & Mamun, M. (2020). Aggregated COVID-19 suicide incidences in India: Fear of COVID-19 infection is the prominent causative factor. *Psychiatry Research, 290*, e113145. 10.1016/j.psychres.2020.113145. 10.1016/j.psychres.2020.113145 PMC7832713 32544650

[CR17] Ethiopia UNHCR Global Focus. (2020). Ethiopia. Retrieved November 28, 2020, https://reporting.unhcr.org/ethiopia

[CR18] Fegert, J. M., Vitiello, B., Plener, P. L., & Clemens, V. (2020). Challenges and burden of the Coronavirus 2019 (COVID-19) pandemic for child and adolescent mental health: A narrative review to highlight clinical and research needs in the acute phase and the long return to normality. *Child and Adolescent Psychiatry and Mental Health, 14*, 20. 10.1186/s13034-020-00329-3. 10.1186/s13034-020-00329-3 PMC7216870 32419840

[CR19] Galea, S., Merchant, R. M., & Lurie, N. (2020). The mental health consequences of COVID-19 and physical distancing: The need for prevention and early intervention. *JAMA Internal Medicine*. 10.1001/jamainternmed.2020.1562. 10.1001/jamainternmed.2020.1562 32275292

[CR20] Geukens, F., Maes, M., Spithoven, A., Pouwels, J. L., Danneel, S., Cillessen, A. H., et al. (2020). Changes in adolescent loneliness and concomitant changes in fear of negative evaluation and self-esteem. *International Journal of Behavioral Development*, 016502542095819. 10.1177/0165025420958194.

[CR21] Griffiths, M. D., & Mamun, M. A. (2020). COVID-19 suicidal behavior among couples and suicide pacts: Case study evidence from press reports. *Psychiatry Research, 289*, 113105. 10.1016/j.psychres.2020.113105. 10.1016/j.psychres.2020.113105 PMC7229970 33242807

[CR22] Hu, L. T., & Bentler, P. M. (1999). Cutoff criteria for fit indexes in covariance structure analysis: conventional criteria versus new alternatives. *Structural Equation Modeling, 6*(1), 1–55. 10.1080/10705519909540118.

[CR23] Information Network Security Agency. (2020). Ethiopia COVID-19 monitoring platform. Retrieved November 15, 2020, from: https://www.covid19.et/covid-19/

[CR24] Jakobsson, U., & Hallberg, I. R. (2005). Loneliness, fear, and quality of life among elderly in Sweden: A gender perspective. *Aging Clinical and Experimental Research, 17*(6), 494–501. 10.1007/BF03327417. 10.1007/BF03327417 16485868

[CR25] Killgore, W., Cloonan, S. A., Taylor, E. C., & Dailey, N. S. (2020). Loneliness: A signature mental health concern in the era of COVID-19. *Psychiatry Research, 290*, 113117. 10.1016/j.psychres.2020.113117. 10.1016/j.psychres.2020.113117 PMC7255345 32480121

[CR26] Kline, R. B. (2015). *Principles and practice of structural equation modeling* (4th ed.). New York: Guilford Publications.

[CR27] Lee, S. A. (2020). Coronavirus Anxiety Scale: A brief mental health screener for COVID-19 related anxiety. *Death Studies**, 44*, 393–401. 10.1080/07481187.2020.1748481. 10.1080/07481187.2020.1748481 32299304

[CR28] Mailliez, M., Griffiths, M. D., & Carre, A. (2020). Validation of the French version of the Fear of COVID-19 Scale and its associations with depression, anxiety and differential emotions. 10.21203/rs.3.rs-46616/v1. 10.1007/s11469-021-00499-x PMC8300066 34335119

[CR29] Mamun, M. A., & Griffiths, M. D. (2020). First COVID-19 suicide case in Bangladesh due to fear of COVID-19 and xenophobia: Possible suicide prevention strategies. *Asian Journal of Psychiatry, 51*, 102073. 10.1016/j.ajp.2020.102073. 10.1016/j.ajp.2020.102073 PMC7139250 32278889

[CR30] Masuyama, A., Shinkawa, H., & Kubo, T. (2020). Validation and psychometric properties of the Japanese version Fear of COVID-19 Scale among adolescents. *Intrnational Journal of Mental Health and Addiction*. 10.31234/osf.io/jkmut. 10.1007/s11469-020-00368-z PMC7358289 32837445

[CR31] Neto, F. (2014). Psychometric analysis of the short-form UCLA Loneliness Scale (ULS-6) in older adults. *European Journal of Ageing, 11*, 313–319. 10.1007/s10433-014-0312-1. 10.1007/s10433-014-0312-1 PMC5549168 28804337

[CR32] Nguyen, H. T., Do, B. N., Pham, K. M., Kim, G. B., Dam, H. T., Nguyen, T. T., Nguyen, T. T., Nguyen, Y. H., Sørensen, K., Pleasant, A., & Duong, T. V. (2020). Fear of COVID-19 Scale—Associations of its scores with health literacy and health-related behaviors among medical students. *International Journal of Environmental Research and Public Health, 17*(11), 4164. 10.3390/ijerph17114164. 10.3390/ijerph17114164 PMC7311979 32545240

[CR33] Oqubay, A. (2020). Adaptive leadership in the Covid-19 response: Insights from Ethiopia. Insight Retrieved June 27, 2020, from: https://www.odi.org/blogs/16974-adaptive-leadership-covid-19-response-insights-ethiopia

[CR34] Pang, N. T. P., Kamu, A., Hambali, N. L. B., Mun, H. C., Kassim, M. A., Mohamed, N. H., Ayu, F., Rahim, S. S. S. A., Omar, A., & Jeffree, M. S. (2020). Malay version of the Fear of COVID-19 Scale: Validity and reliability. *International Journal of Mental Health and Addiction*. 10.1007/s11469-020-00355-4. 10.1007/s11469-020-00355-4 PMC7333973 32837437

[CR35] Reardon, S. (2015). Ebola’s mental-health wounds linger in Africa. *Nature, 519*(7541), 13–14. 10.1038/519013a. 10.1038/519013a 25739606

[CR36] Reznik, A., Gritsenko, V., Konstantinov, V., Khamenka, N., & Isralowitz, R. (2020). COVID-19 fear in Eastern Europe: Validation of the Fear of COVID-19 Scale. *International Journal of Mental Health and Addiction*. 10.1007/s11469-020-00283-3. 10.1007/s11469-020-00283-3 PMC7217343 32406404

[CR37] Rossi, A., Panzeri, A., Pietrabissa, G., Manzoni, G. M., Castelnuovo, G., & Mannarini, S. (2020). The anxiety-buffer hypothesis in the time of COVID-19: when self-esteem protects from the impact of loneliness and fear on anxiety and depression. *Frontiers in Psychology, 11*, 2177. 10.3389/fpsyg.2020.02177. 10.3389/fpsyg.2020.02177 PMC7683508 33240140

[CR38] Russell, D., Peplau, L. A., & Cutrona, C. E. (1980). The revised UCLA Loneliness Scale: Concurrent and discriminant validity evidence. *Journal of Personality and Social Psychology, 39*(3), 472–480. 10.1037/0022-3514.39.3.472. 10.1037//0022-3514.39.3.472 7431205

[CR39] Sakib, N., Bhuiyan, A. I., Hossain, S., Al Mamun, F., Hosen, I., Abdullah, A. H., et al. (2020). Psychometric validation of the Bangla Fear of COVID-19 Scale: Confirmatory factor analysis and Rasch analysis. *International Journal of Mental Health and Addiction*. 10.1007/s11469-020-00289-x. 10.1007/s11469-020-00289-x PMC7213549 32395096

[CR40] Satici, B., Gocet-Tekin, E., Deniz, M. E., & Satici, S. A. (2020). Adaptation of the Fear of COVID-19 Scale: Its association with psychological distress and life satisfaction in Turkey. *International Journal of Mental Health and Addiction*. 10.1007/s11469-020-00294-0. 10.1007/s11469-020-00294-0 PMC7207987 32395095

[CR41] Soraci, P., Ferrari, A., Abbiati, F. A., del Fante, E., de Pace, R., Urso, A., & Griffiths, M. D. (2020). Validation and psychometric evaluation of the Italian version of the Fear of COVID-19 Scale. *International Journal of Mental Health and Addiction*. 10.1007/s11469-020-00277-1. 10.1007/s11469-020-00277-1 PMC7198091 32372892

[CR42] Taylor, S., Landry, C., Paluszek, M., Fergus, T. A., McKay, D., & Asmundson, G. J. (2020). Development and initial validation of the COVID Stress Scales. *Journal of Anxiety Disorders, 72*, 102232. 10.1016/j.janxdis.2020.102232. 10.1016/j.janxdis.2020.102232 PMC7198206 32408047

[CR43] Thakur, V., & Jain, A. (2020). COVID 2019-suicides: A global psychological pandemic. *Brain, Behavior, and Immunity, S0889-1591*(20), 30643-7. 10.1016/j.bbi.2020.04.062. 10.1016/j.bbi.2020.04.062 PMC7177120 32335196

[CR44] Tsipropoulou, V., Nikopoulou, V. A., Holeva, V., Nasika, Z., Diakogiannis, I., Sakka, S., Kostikidou, S., Varvara, C., Spyridopoulou, E., & Parlapani, E. (2020). Psychometric properties of the Greek version of FCV-19S. *International Journal of Mental Health and Addiction*. 10.1007/s11469-020-00319-8. 10.1007/s11469-020-00319-8 PMC7250285 32837420

[CR45] Whitehead, E. E., & Whitehead, J. D. (2010). *Transforming our painful emotions: spiritual resources in anger, shame, grief, fear and loneliness*. Maryknoll: Orbis Books.

[CR46] Winter, T., Riordan, B. C., Pakpour, A. H., Griffiths, M. D., Mason, A., Poulgrain, J. W., & Scarf, D. (2020). Evaluation of the English version of the Fear of COVID-19 Scale and its relationship with behavior change and political beliefs. *International Journal of Mental Health and Addiction*. Advance online publication. 10.1007/s11469-020-00342-9. 10.1007/s11469-020-00342-9 PMC7295324 32837431

[CR47] World Bank. (2019). *Ethiopia gender diagnostic report: Priorities for promoting equity*. World Bank.Retrieved November 28, 2020, from: http://hdl.handle.net/10986/31420.

[CR48] World Health Organization. (2020a). WHO Coronavirus Disease (COVID-19) Dashboard Coronavirus disease (COVID-19) outbreak. Retrieved November 28, 2020, from: https://covid19.who.int/

[CR49] World Health Organization (2020b). Mental health and COVID-19. Retrieved June 30, 2020, from: https://www.euro.who.int/en/health-topics/health-emergencies/coronavirus-covid-19/technical-guidance/mental-health-and-covid-19

[CR50] Worldometers (2020). Daily New COVID-19 Cases in Ethiopia. Retrieved November 15, 2020, https://www.worldometers.info/coronavirus/country/ethiopia/

